# The Use of Social Media to Express and Manage Medical Uncertainty in Dyskeratosis Congenita: Content Analysis

**DOI:** 10.2196/46693

**Published:** 2024-01-15

**Authors:** Emily Pearce, Hannah Raj, Ngozika Emezienna, Melissa B Gilkey, Allison J Lazard, Kurt M Ribisl, Sharon A Savage, Paul KJ Han

**Affiliations:** 1 Division of Cancer Epidemiology and Genetics, Clinical Genetics Branch National Cancer Institute National Institutes of Health Rockville, MD United States; 2 Team Telomere Coeur d'Alene, ID United States; 3 Department of Health Behavior Gillings School of Global Public Health University of North Carolina at Chapel Hill Chapel Hill, NC United States; 4 Hussman School of Journalism and Media University of North Carolina at Chapel Hill Chapel Hill, NC United States; 5 Division of Cancer Control and Population Sciences, Behavioral Research Program National Cancer Institute National Institutes of Health Rockville, MD United States

**Keywords:** social media, medical uncertainty, telomere biology disorder, dyskeratosis congenita, social support

## Abstract

**Background:**

Social media has the potential to provide social support for rare disease communities; however, little is known about the use of social media for the expression of medical uncertainty, a common feature of rare diseases.

**Objective:**

This study aims to evaluate the expression of medical uncertainty on social media in the context of dyskeratosis congenita, a rare cancer-prone inherited bone marrow failure and telomere biology disorder (TBD).

**Methods:**

We performed a content analysis of uncertainty-related posts on Facebook and Twitter managed by Team Telomere, a patient advocacy group for this rare disease. We assessed the frequency of uncertainty-related posts, uncertainty sources, issues, and management and associations between uncertainty and social support.

**Results:**

Across all TBD social media platforms, 45.98% (1269/2760) of posts were uncertainty related. Uncertainty-related posts authored by Team Telomere on Twitter focused on scientific (306/434, 70.5%) or personal (230/434, 53%) issues and reflected uncertainty arising from probability, ambiguity, or complexity. Uncertainty-related posts in conversations among patients and caregivers in the Facebook community group focused on scientific (429/511, 84%), personal (157/511, 30.7%), and practical (114/511, 22.3%) issues, many of which were related to prognostic unknowns. Both platforms suggested uncertainty management strategies that focused on information sharing and community building. Posts reflecting response-focused uncertainty management strategies (eg, emotional regulation) were more frequent on Twitter compared with the Facebook community group (χ^2^_1_=3.9; *P*=.05), whereas posts reflecting uncertainty-focused management strategies (eg, ordering information) were more frequent in the Facebook community group compared with Twitter (χ^2^_1_=55.1; *P*<.001). In the Facebook community group, only 36% (184/511) of members created posts during the study period, and those who created posts did so with a low frequency (median 3, IQR 1-7 posts). Analysis of post creator characteristics suggested that most users of TBD social media are White, female, and parents of patients with dyskeratosis congenita.

**Conclusions:**

Although uncertainty is a pervasive and multifactorial issue in TBDs, our findings suggest that the discussion of medical uncertainty on TBD social media is largely limited to brief exchanges about scientific, personal, or practical issues rather than ongoing supportive conversation. The nature of uncertainty-related conversations also varied by user group: patients and caregivers used social media primarily to discuss scientific uncertainties (eg, regarding prognosis), form social connections, or exchange advice on accessing and organizing medical care, whereas Team Telomere used social media to express scientific and personal issues of uncertainty and to address the emotional impact of uncertainty. The higher involvement of female parents on TBD social media suggests a potentially greater burden of uncertainty management among mothers compared with other groups. Further research is needed to understand the dynamics of social media engagement to manage medical uncertainty in the TBD community.

## Introduction

### Background

Medical uncertainty is a common experience in rare diseases and may combine with limited scientific knowledge and access to peer groups to impede a patient’s ability to seek and adhere to medical treatments [1] and intensify health-related anxiety, decreasing quality of life for patients and their caregivers [2,3]. Dyskeratosis congenita (DC) is a rare telomere biology disorder (TBD) associated with very high risks of bone marrow failure, pulmonary and liver disease, cancer, and other medical conditions. Diagnosis is challenging because of its wide phenotypic spectrum, including the classic DC triad (nail dysplasia, abnormal skin pigmentation, and oral leukoplakia) with pediatric bone marrow failure, middle-age presentation with pulmonary failure or aplastic anemia, abnormally short telomere length, or detection of pathogenic germline variants in >18 different genes [4]. Although age of onset is variable, DC often presents in childhood and adolescence, with most patients experiencing their first symptoms before the age of 20 years [5]. Diagnosis frequently results in a lifetime commitment to screening to detect progressive clinical manifestations of DC, including cancers across multiple organ systems [5]. Owing to the complexity and rarity of DC and related TBDs, patients and their families often have long diagnostic journeys, face complicated health decision-making, and frequently do not have access to medical professionals and supportive peers who are familiar with their condition. This situation likely creates a substantial burden of medical uncertainty for patients with TBDs and their families. Although medical uncertainty has been associated with increased anxiety and difficulty with decision-making in rare diseases and cancer occurrence and recurrence [6-11], to date, no research has addressed the experience or management of medical uncertainty in the TBD context.

As outlined in a previously published taxonomy developed by Han [12], uncertainty in medicine arises from multiple sources (eg, probability, ambiguity, and complexity) and focuses on scientific, personal, and practical issues. These situations activate a variety of management strategies to address uncertainty, which are primarily cognitive, emotional, and relational in nature. Uncertainty management strategies may target ≥1 sources or issues of uncertainty and are defined as belonging to ≥1 of the following approaches: seeking information to fill knowledge gaps (“ignorance-focused”), reducing or increasing attention to unknowns (“uncertainty-focused”), ameliorating adverse psychological effects of uncertainty (“response-focused”), and fostering interpersonal relationships to engage with uncertainty as a shared experience (“person-focused”). In situations where uncertainty cannot be reduced, these strategies may mitigate its negative mental health impact and help individuals achieve an adaptive, optimal balance of responses to uncertainty (uncertainty tolerance).

The rarity of TBDs suggests a potential role for internet-based platforms to deliver social support by bridging geographic, knowledge, and community network limitations. Social support, a complex concept encompassing a variety of helping social interactions [13], includes four main types: (1) expression of empathy and care (emotional), (2) provision of tangible assistance (instrumental), (3) provision of knowledge or facts (informational), and (4) evaluative feedback about task performance or personal qualities (appraisal) [14]. Research suggests that social support decreases the experience of stress, anxiety, and depression and improves the overall quality of life in populations experiencing medical uncertainty [8,10,15-17]. The benefit of social support has been demonstrated in patients with Li-Fraumeni syndrome, a rare genetic cancer predisposition, where informational, tangible, spiritual, and emotional support from in-person sources enhanced positive coping capacities [18]. Social media platforms such as Facebook and Twitter have been identified as important resources for social support in rare disease contexts [19-24], and disease-specific social media support has been recommended in oncology [25], rare genetic disease [26-28], and other stigmatized or rare diseases [29-31]. In addition to increasing access to information and social networks, continued participation in socially supportive internet-based communities may also build capacities for uncertainty tolerance [10,17,32-38]. Although social media has the potential to bridge geographic or social boundaries, its use is often concentrated in select populations, limiting its reach and potentially inhibiting its use by some groups [39,40]. In addition, dynamics observed on social media posts may not reflect real-life experiences and are limited in depth and detail, increasing the potential for misinterpretation [39]. Social media can also spread misinformation with damaging consequences, especially in high-uncertainty health contexts [41-43].

### Objectives

Although extensive research has investigated the psychosocial benefits of internet-based health forums for patients and their caregivers [23,28,29,44-51], there is still a need to evaluate the use of social media to express or manage medical uncertainty in rare diseases. Specifically, we need to examine social media use for expressing and managing medical uncertainty in TBDs to understand the experience of medical uncertainty in this context and to build evidence to improve health communication and uncertainty management interventions [52]. This exploratory study aims to review social media posts created by and targeted at patients with TBDs and their caregivers to (1) measure the frequency of uncertainty-related posts; (2) catalog the issues, sources, and types of uncertainty and uncertainty management strategies; (3) measure user engagement with different post types; and (4) explore the relationship between uncertainty and social support. To achieve these aims, we reviewed all publicly available social media sites owned and maintained by Team Telomere (previously DC Outreach, Inc), the oldest and largest patient advocacy organization for individuals, caregivers, and families affected by TBDs worldwide [53]. The social media of Team Telomere constitutes the most expansive and accessible body of internet-based TBD-related content, inclusive of a variety of user perspectives. The variety of posts by users with diverse connections to TBDs (eg, medical providers, patients, caregivers, and health advocacy nonprofits) makes Team Telomere’s social media an ideal data source for understanding the range and dynamics of medical uncertainty communication and social support exchange in the TBD context.

## Methods

### Ethical Considerations

Data collection was undertaken in partnership with Team Telomere following best practices guidelines for social media research [54] and was approved by the National Institutes of Health Institutional Review Board (IRB 000722).

### Data Source

The source of data for this study was all publicly available social media owned and maintained by Team Telomere. These sites included the Team Telomere Twitter page [55], the Facebook main page [56], and a public Facebook community group [57] (Table 1). All the sites were open to the public and had no eligibility requirements for membership. Content across all platforms was monitored by Team Telomere to ensure appropriate adherence to community guidelines, and Team Telomere’s staff removed posts with offensive or scientifically inaccurate content. The Facebook main page and Twitter accounts were created to promote the work of Team Telomere “supporting families worldwide affected by Dyskeratosis Congenita and Telomere Biology Disorders” [56]. The Facebook community group was created in response to social isolation following the COVID-19 pandemic as “a place to share our everyday lives in the spirit of promoting and maintaining connections among our Team Telomere/Dyskeratosis Congenita/Telomere Biology disorder community” [57].

**Table 1:** Data source characteristics at the time of the study.

	Facebook community group	Facebook main page	Twitter
Creation date (y)	2020	2010	2010
Followers, n	187	1637	1933
Posts^a^, n	511	1815	434

^a^Represents posts captured during the study period (June 2019 to December 2021).

### Inclusion

All posts made on Team Telomere’s social media (Facebook main page: n=1818, Facebook community group: n=518, and Twitter: n=441) between June 2019 and December 2021 were eligible for inclusion. This time frame encompasses the period starting 1 year before the Facebook community group. This group was created in June 2020 as a platform for social connection during the COVID-19 pandemic. Posts were excluded from the analysis if they were (1) removed by the user or Team Telomere (n=5), (2) duplicate posts with identical content from the same day (n=2), or (3) posts without image or text content (n=7). This resulted in a total of 2760 posts, with both primary posts and comments considered unique. The post was used as the unit of analysis and included all content visible to a passive social media user. Additional post content that required clicking links to external sites or embedded audiovisual materials was not included in this study.

### Data Extraction and Quality Control

We met with Team Telomere’s leadership (eg, executive director and board) before conducting the study and cocreated a community-based research contract outlining parameters. Although all data were publicly available and Facebook data were manually extracted by the authors, Team Telomere facilitated data extraction from Twitter by sharing downloaded images and text files made available to them as account owners. We used the post (original or responses), rather than post creator, as the unit of measurement and did not collect identifying information of the social media users or interact directly with users.

Data were extracted directly from each social media site manually through (1) screenshots saved as deidentified image files and (2) cut-and-paste of post text into an Excel (Microsoft Corporation) spreadsheet. For the Facebook community group, we assigned unique ID numbers to post creators using public data (usernames) to calculate how many unique users engaged in conversation threads, and we viewed the publicly available profile images to assess observed sex and race. Posts were assigned a unique ID number within Excel, and additional data were manually extracted for each post to capture the post popularity (number of likes, shares, and comments), post type (primary post or comment), and types of emojis present. Demographics of post creators (observed gender and race) were assessed through an independent review of profile images and profile names by 3 coders (EP, HR, and NE). Quality control for data extraction was performed on a subset of the data (n=100 posts) by NE, and intercoder reliability was assessed during the multiple-reviewer coding process.

### Coding and Analysis

We used a combined content analysis mixed methods approach to analyze the social media data [58]. This involved qualitative analysis (coding by multiple independent reviewers) and quantitative analysis (frequency and chi-square testing). Constructs were defined through codebook development using deductive (theory driven) approaches, whereas qualitative themes were identified through inductive (data driven) discussion, as described in greater detail in the *Methods* section. The analysis was performed separately for each social media source, 2 Facebook pages (the Team Telomere main page and a separate community group page established in 2020) and the Team Telomere Twitter feed, resulting in the creation of 3 separate data sets (Facebook main page: n=1815, Facebook community group: n=511, and Twitter: n=434). A subset of Facebook community group posts (n=77; 12 primary posts and 65 comments) was reviewed by 3 coders and used to inform uncertainty inclusion criteria (Multimedia Appendix 1) and the codebook (Multimedia Appendix 2) developed to deductively identify the presence or absence of uncertainty and social support constructs defined in the Han Taxonomy of Medical Uncertainty [12] and the Social Support Framework [14]. Then, all posts were coded for uncertainty and social support by 3 independent coders (EP, HR, and PKJH), with all disagreements in coding resolved through discussion and consensus. Posts identified as uncertainty related in the Facebook community group (n=156) and Twitter (n=210) were then independently subcoded (EP, HR, and PKJH) for uncertainty issues, sources, and management strategies according to the codebook definitions detailed in the *Measures* section. Data were then arranged by subcode and reviewed qualitatively to detect themes that emerged from the data and were refined through discussion between coders.

### Measures

#### Intercoder Reliability

Intercoder reliability among the 3 coders was measured across all social media types for the initial coding of dichotomous social support and uncertainty variables using Cohen κ. The analysis found acceptable reliability of independent coders in assessing the presence or absence of any social support (κ value range across all platforms, κ=0.79-0.95) and uncertainty (κ value range across all platforms, κ=0.58-0.93) across all social media platforms. Regardless, all discrepancies were mutually resolved through coder consensus.

#### Post Creator Characteristics

Post creator characteristics were visible from profile images and usernames that appeared alongside each post. Posts from Team Telomere’s organizational account were created by staff members, often identified in the post context (eg, executive director, communications director, or board member). We did not scrutinize user profiles to detect the activity of nonhuman bots; however, in the context of the small population with this rare disease, most users could be positively identified as human beings from the context of their posts and history of participation in organizational events. Post creator characteristics, including observed gender and race, were assessed by 3 independent coders’ perceptions of publicly available usernames and profile images. Disagreements between coders resulted in the characteristic being coded as “unknown.”

#### Uncertainty Issues, Sources, and Management Strategies

Posts were coded as uncertainty related according to 1 of the following types: (1) uncertainty-related primary posts, (2) uncertainty-related comments, and (3) non–uncertainty-related posts captured within a thread where 1 or more other post was uncertainty related. For the Facebook community group and Twitter, posts identified as uncertainty-related primary posts or comments were further analyzed to determine the presence or absence of sources (ambiguity, complexity, and probability), issues (scientific, personal, and practical), and attributes of uncertainty management strategies (ignorance focused, uncertainty focused, response focused, and person focused). We defined sources of uncertainty as insufficient, unreliable, or contradictory information (ambiguity); information features, such as multiple or interacting causes and effects that make a phenomenon difficult to understand (complexity); and fundamental randomness or indeterminacy of a phenomenon that makes outcomes unpredictable (probability). We defined issues of uncertainty as pertaining to the causes, diagnosis, prognosis, or management of disease (scientific); the impact of disease on aspects of personal life (personal); and logistical issues related to health care or disease management (practical). Although the data did not allow assessment of intent to manage uncertainty, we searched posts to identify evidence of management strategies with ≥1 of the following attributes: (1) providing or seeking information to fill knowledge gaps (ignorance focused), (2) reducing or increasing attention to unknowns to gain or relinquish a sense of control (uncertainty focused), (3) ameliorating the adverse psychological effects of uncertainty (response focused), and (4) fostering interpersonal relationships to engage with uncertainty as a shared experience (person focused).

#### Social Support

Posts were categorized as containing social support through qualitative coding by 3 independent reviewers (EP, HR, and PKJH) following definitions developed over decades of research in social support theory [14,59,60]. Dichotomous variables were assigned to indicate the presence or absence of social support and the presence or absence of specific types of support within 4 domains (appraisal, emotional, informational, and instrumental). These domains were defined as (1) giving or receiving evaluative feedback (appraisal); (2) giving or receiving indicators of care, love, appreciation, empathy, or sympathy (emotional); (3) giving or receiving knowledge (informational); and (4) giving or receiving tangible support (instrumental), as recently formulated by Holt-Lunstad and Uchino [14]. Assignment to social support domains was not mutually exclusive.

#### Relationship Between Social Support and Uncertainty

We examined the relationship between social support and uncertainty by comparing frequencies and chi-square tests. Posts were coded as dichotomous variables for uncertainty (uncertainty related, non–uncertainty related), uncertainty subtypes (presence or absence), and social support subtypes (presence or absence). We examined the frequencies of social support subtypes in uncertainty-related posts overall, by social media platform (Facebook community group and Twitter) and by post type (primary post or comment). We performed chi-square tests to determine the strength of the relationship between uncertainty-related posts and social support across platforms and for uncertainty-related posts by post type (primary post, comment, thread) and issue subtype (scientific, personal, practical).

#### Popularity and Engagement

Popularity on the Facebook community group, Facebook main page, and Twitter was defined as the sum of comments, likes, and shares. Engagement was defined separately for social media types (Facebook community group and Facebook main page vs Twitter) owing to differences in user tracking approaches between Facebook and Twitter platforms. Facebook engagement was defined as the sum of conversations (number of responses generated by a post or comment), voices (number of unique users responding to a post or comment), and depth (number of back-and-forth responses). Engagement on Twitter was defined as the sum of detail expands (clicks to view more of the post), profile visits, link clicks, and video views. Engagement was also measured for the Facebook community group by examining the proportion of users who contributed posts and post frequencies by author.

#### Sentiment

Sentiment analysis was performed through manual annotation by 2 independent coders, with differences resolved through consensus. Posts were assigned categorical sentiment variables according to the (1) frequency and (2) presence or absence of keywords and emojis. Unambiguous emotion words (eg, “happy” and “sad”) were chosen as keywords to indicate emotional valence, as described in other studies [61,62]. The emotional valence of emojis was assigned based on the emoji definition in internet-based emoji dictionaries and validated by a coder review of the emoji within the post context (Multimedia Appendix 3).

## Results

### Post Characteristics

A total of 2760 posts created on all platforms between June 2019 and December 2021 were included in this study. Across all platforms, most posts were created either by the executive director of Team Telomere or by individual users who were primarily identified as White, female, and parents of children affected by TBDs. Post characteristics differed by platform: on Twitter, most posts (368/434, 84.8%) were primary posts, most of which (384/434, 88.5%) were generated by the executive director of Team Telomere; Facebook main page posts were either primary posts (800/1815, 44.08%) or first-level comments (1014/1815, 55.87%) created by Team Telomere (860/1815, 47.38%) or individual users (955/1815, 52.62%); and on the Facebook community group, most posts (403/511, 78.9%) were comments to primary posts, in sometimes lengthy (up to 8 level) conversation threads created by 67 individual users (502/511, 98.2%). Posts across all platforms were written almost exclusively in English (Table 2).

**Table 2:** Characteristics of posts on Team Telomere’s social media from June 2019 to December 2021 (N=2760).

		Facebook community group (n=511), n (%)	Facebook main page (n=1815), n (%)	Twitter (n=434), n (%)
**Post type**				
	Primary post	108 (21.1)	800 (44.1)	368 (84.8)
	Comment	403 (78.9)	1015 (55.9)	66 (15.2)
**Language**				
	English	487 (95.3)	1807 (99.6)	434 (100)
	Other^a^	4 (0.8)	8 (0.4)	0 (0)
	Image only	17 (3.3)	0 (0)	0 (0)
**Creator type**				
	Team telomere	8 (1.6)	861 (47.4)	385 (88.7)
	Individual	503 (98.4)	954 (52.6)	49 (11)
**Observed creator sex^b^**				
	Male	25 (5)	69 (7.2)	5 (10)
	Female	478 (95)	885 (92.8)	41 (83.7)
	Unknown	0 (0)	1 (0.1)	3 (6.1)
**Observed creator race^b^**				
	White	443 (88.1)	766 (80.3)	40 (81.6)
	Other^c^	46 (9.1)	30 (3.1)	6 (12.2)
	Unknown	14 (2.8)	158 (16.6)	3 (6.1)
**Observed creator telomere biology disorder relationship^b,d^**				
	Patient	65 (12.9)	42 (4.4)	1 (2)
	Parent	428 (85.1)	384 (40.3)	14 (28.6)
	Medical provider	3 (0.6)	31 (3.2)	10 (20.4)
	Other^e^	5 (1)	59 (6.2)	22 (44.9)
	Unknown	40 (8)	495 (51.9)	2 (4.1)
	Multiple	129 (25.6)	126 (13.2)	0 (0)

^a^Respectively by platform (Facebook community group, Facebook main page, and Twitter), “other” language included Spanish (0.2%, 0.2%, and 0%), French (0.4%, 0.1%, and 0%). In the Facebook community group the following languages also appeared: Hebrew (0.1%), Italian (0.1%), Swedish (0.1%), and Māori (0.2%).

^b^Includes frequencies for individual creator types only; does not include Team Telomere organization (Facebook community group: n=503, Facebook main page: n=954, and Twitter: n=49).

^c^Respectively by platform (Facebook community group, Facebook main page, and Twitter), “other” identified creator race and ethnicity included Latinx (7.7%, 1.5%, and 1.4%) and Arab or Middle Eastern (1.4%, 11%, and 0%).

^d^Frequency does not total to 100% because of some individuals occupying multiple categories.

^e^Respectively by platform (Facebook community group, Facebook main page, and Twitter), “other” creator telomere biology disorder relationship included grandparent (0%, 0.2%, and 0%), sibling (0.4%, 0.9%, and 0%), spouse (0%, 0.2%, and 0%), other advocacy organization representative (not Team Telomere; 0%, 0%, and 40.8%), and clinical or pharmaceutical industry representative (0%, 0.1%, and 4.1%).

### Qualitative Findings

Qualitative analysis of posts revealed multiple uncertainty issues, sources, and management indicators. Issues included diagnostic, prognostic, therapeutic, and causal uncertainties (scientific); assembly of medical care teams, geographic or financial constraints, and limitations to research funding and dissemination (practical); and building “rare” identity, communicating complex health information to children, and reframing educational or developmental goals (personal). Sources of uncertainty included confusing symptoms and lack of clarity in medical advice (ambiguity); the TBD impact of TBD on multiple organ systems, managing medications or screening regimens, emotional confusion, and achieving scientific literacy across different medical specialties (complexity); and prognostic outcomes, behavioral health risks, or genetic inheritance (probability). Attributes of uncertainty management strategies included (1) information seeking, participation in research, and connection to trusted information sources and care providers (ignorance focused); (2) ordering multiple uncertainties through categorization, prioritization, and sequential narratives, including counting of survival days since transplant (uncertainty focused); (3) sharing positive emotions, portraying TBD experience as a source of strength, and encouraging relaxation (response focused); and (4) promoting a TBD community identity by creating a community mascot (a unicorn named “Tillymere”), recognizing community-specific celebrations (TBD month and transplant anniversaries), providing TBD-pride identifiers (T-shirts and swag), and making reference to Team Telomere as a “family” (person focused; Table 3).

**Table 3:** Uncertainty in telomere biology disorder (TBD) social media.

		Post text
**Sources of uncertainty**		
	Ambiguity	“This is a tough one! One of those maybe/maybe not symptoms...I often ask myself the same questions about my daughter’s more obscure symptoms.” [FBCG218304.21.07.30] “Pre-lung # transplantation patients with # pulmonary # fibrosis who have short # telomeres may need different # clinical care...” [TWT180100.19.06.11]
	Complexity	“[Name] is having kidney, heart, and lung problems. Oh, and who can forget the liver? This week has been too long at the hospital” [FBCG2110000.21.11.23] “# DYK Those with # telomere biology disorders may be especially vulnerable to the effects of taking multiple medicines at the same time and may respond to medications differently.” [TWT186700.19.11.14]
	Probability	“80% of patients diagnosed with dyskeratosis congenita will experience bone marrow failure.” [TWT185500.19.11.04] “5 out of 6 of the cell lines tested were less than 1%. And when that’s the case, patients have a 10-20% chance of getting cancer...” [FBCG203500.20.09.08] “A recent publication advises against an elective eye surgery in patients with DC due to higher long-term risks caused by delayed healing...” [TWT182100.19.08.25]
**Issues of uncertainty**		
	Scientific	“Has anybody experienced hearing loss with connection to short telomere length?” [FBCG218300.21.07.30] “Has anyone had kidney problems outside of BMT? Are there any articles anyone has seen on kidneys and short telomeres?” [FBCG2110000.21.11.23]
	Practical	“At the moment [Name] has 1-2 appointments each week. Add to that emails to/from paediatrician, calls from hospital to change/confirm appointments...It’s overwhelming some weeks. And I’m usually doing all this from work. We are also applying for different supports...so lots of forms, phone calls and emails!” [FBCG204305-8.20.10.13]
	Personal	“It’s # PFMonth, and we want you to know you have a team surrounding you...” [TWT1816000.20.09.04] “TBDs are not just a pediatric disease.Affected adults with a # raredisease, you are NOT ALONE!” [TWT183100.19.09.21] “Another milestone reached. This time five years ago as we celebrated [Name]’s 5th birthday we were also getting ready to go to transplant two weeks later. Yesterday we celebrated the big 10...” [FBCG201300.20.06.27]
**Focus of uncertainty management**		
	Ignorance	“Wondering if anyone with DC had a dental implant post-transplant...? And did your medical team have any concerns or recommendations?” [FBCG215500.21.01.05] “Hello—any contraindications to getting COVID 19 vaccine if you have DC?” [FBCG217100.21.04.04] “Do you have a copy of the clinical guidelines?” [FBCG203509.20.09.08] “Take time to learn more about #Telomere Biology Disorders through our informational video!” [TWT1822100.21.11.04]
	Uncertainty	“Each Family Story is set up so you can find a connection via gene or experience.” [FBCG204400.20.10.29] “My daughter has yearly bone marrow biopsies, lung and liver screenings. ENT and skin checks for cancer.” [FBCG 203513.20.09.08] “I’ve been preparing something for the new school trying to give them what her medical challenges are.” [FBCG219900.21.11.16]
	Response	“Our family is celebrating today! [Name]’s Happy 8th bone marrow transplant anniversary!” [FBCG203300.20.08.24] “Fitting for us all: it wasn’t the trauma that made you strong, kinder, and more compassionate. It’s how you handled it. That credit is yours.” [FBCG216200.21.02.28] “Join@sixnwstevies as she teaches yoga for research... 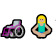 ” [TWT1822600.21.03.16]
	Person	“Thank goodness for social media otherwise it would be very isolating.” [FBCG203821.20.09.25] “Don’t forget to register for our Young Adult Meetup...” [TWT1814300.20.06.23] “[Name] it’s never ending, I hope you find a way to take care of you” [FBCG204307.20.10.13] “You are in great hands but always happy to connect with [Provider Name]” [FBCG203504.20.09.08] “Check out # tillymere! All # sparkly and ready for # TBDmonth!” [TWT185400.19.11.04] “We have all known the long loneliness and we have learned that the only solution is love and that love comes with community. – Dorothy Day” [TWT1816300.20.09.12]

### Uncertainty Issues, Sources, and Management Strategies

Content analysis revealed that 45.98% (1269/2760) of posts overall were uncertainty related, although the frequency differed by platform (Facebook main page: 691/1715, 40.29%; Facebook community group: 155/511, 30.3%; and Twitter: 210/434, 48.4%). Most uncertainty-related posts on Facebook community group and Twitter were generated by Team Telomere’s organizational profile (332/511, 65% and 353/434, 81.3%, respectively) and were often similar in topic, wording, and image content. In the Facebook community group, all uncertainty-related posts were generated by individual users, including a portion (119/511, 23.3%) posted by Team Telomere–affiliated volunteer group moderators.

Owing to low frequency of community-generated uncertainty content on the Facebook community group and Twitter, compared with the Facebook community group, we decided to code uncertainty subtypes only within the Facebook community group and Twitter to compare how medical uncertainty was expressed on social media by 2 contrasting content creator groups (community members vs advocacy organization). Scientific uncertainty was the most common issue on both platforms (305/434, 70.3% to 429/511, 84%). On Twitter, personal uncertainty was more frequently discussed, whereas in the Facebook community group, practical uncertainty was more frequent. Across platforms, most posts (1713/2760, 62.07%) had multiple sources of uncertainty, and a substantial number of posts (1126/2760, 40.8%) were coded as emerging from the combined information features of probability, complexity, and ambiguity. The most common attributes of uncertainty management styles detected on both platforms were requests or offers of information to fill knowledge gaps (ignorance focused) and offers of emotional support or community building (person focused). Response-focused management style attributes (eg, yoga and meditation classes) were marginally more frequent on Twitter compared with the Facebook community group (χ^2^_1_=3.9; *P*=.05), but on the Facebook community group, indicators of uncertainty-focused management (eg, strategies for organization of care logistics) were more frequent compared with Twitter (χ^2^_1_=55.1; *P*<.001; Table 4).

**Table 4:** Characteristics and frequency of uncertainty-related posts on Team Telomere’s Facebook community group and Twitter (N=2760).

		Facebook community group (n=156), n (%)	Twitter (n=210), n (%)	Chi-square (*df*)^a^	*P* value	
**Issue**						
	Personal	48 (30.8)	111 (52.9)	16.6 (1)	<.001	
	Practical	35 (22.4)	23 (11)	9.2 (1)	.002	
	Scientific	131 (84)	148 (70.5)	11.4 (1)	.007	
	Multiple	53 (34)	59 (28.1)	—^a^	—	
**Source**						
	Ambiguity	81 (51.9)	80 (38.1)	17.6 (1)	<.001	
	Complexity	81 (51.9)	75 (35.7)	20.8 (1)	<.001	
	Probability	112 (71.8)	81 (38.6)	71.3 (1)	<.001	
	Multiple	88 (56.4)	77 (36.7)	—	—	
**Management attributes**						
	Ignorance focused	124 (79.5)	156 (74.3)	1.9 (1)	.16	
	Person focused	106 (67.9)	125 (59.5)	3.6 (1)	.06	
	Response focused	57 (36.5)	100 (47.6)	3.9 (1)	.05	
	Uncertainty focused^b^	53 (34)	10 (4.8)	55.1 (1)	<.001	
	Multiple	106 (67.9)	131 (62.4)	—	—	

^a^Chi-square tests were not performed for issues, sources, or management attributes assigned to multiple categories.

^b^Uncertainty thread includes non–uncertainty-related posts captured in a thread where ≥1 other posts were uncertainty related.

### Facebook Social Support and Uncertainty

Frequent overlap of social support and uncertainty was found across all platforms, with uncertainty-related posts being more likely to contain social support compared with non–uncertainty-related posts (χ^2^_1_=70.7; *P*<.001). However, within social support subtypes, only informational support remained significantly more frequent within uncertainty-related posts (χ^2^_1_=486.0; *P*<.001), whereas emotional support was significantly less frequent in uncertainty-related posts (χ^2^_1_=66.5; *P*<.001) compared with non–uncertainty-related posts. The relationship between informational support and uncertainty remained significant for all social media types, but the relationship between emotional support and uncertainty differed by platform (Multimedia Appendix 4). Emotional support was significantly more frequent in uncertainty-related posts for the Facebook community group (χ^2^_1_=7.8; *P*=.005), was significantly less frequent in uncertainty-related posts on the Facebook main page (χ^2^_1_=79.5; *P*<.001), and had no relationship with uncertainty-related posts on Twitter (χ^2^_1_=0.5; *P*=.47).

On all platforms, uncertainty-related posts were more frequently offers of support than requests. When requests occurred, they were more likely to appear on the Facebook community group compared with Twitter (*χ*^2^_1_=12.7; *P*<.001). Posts that were not uncertainty related but appeared in an uncertainty-related thread frequently contained offers of emotional support.

Given the greater variation in types and direction (offer vs request) of social support in the Facebook community group, we decided to focus on subsequent analyses of the relationship between social support and uncertainty subtypes on this platform. Analysis of social support in the Facebook community group posts by uncertainty issue found that informational support was offered more frequently in response to scientific and practical uncertainty posts compared with personal uncertainty posts. Informational support was also the most frequent type of support requested and offered across uncertainty source types in the Facebook community group; however, uncertainty posts emerging from probability concerns had similar frequencies of emotional and informational support (320/511, 62.6% and 511/836, 61.1%, respectively). This was particularly true in the case where a post had multiple uncertainty sources, which were more likely to be coded as informational support offers or requests compared with posts with only a single uncertainty source (χ^2^_1_=90.4; *P≤*.001).

### Popularity and Engagement

Popularity and engagement were positively skewed toward lower values across all social media types. Popularity was highest for posts on Twitter (Facebook community group: median 1, range 0-55, mean 4, SD 7.5; Facebook main page: range 0-151, median 1, mean 5.9, SD 13.3; and Twitter: range 0-1147, median 13, mean 28.8, SD 76.6). However, engagement was higher in the Facebook community group than on the Facebook main page or Twitter (Facebook community group: range 0-29.6, median 0.54, mean 2.15, SD 4.0; Facebook main page: median 0.0006, range 0-0.09, mean 0.004, SD 0.008; and Twitter: median 0.007, range 0-0.56, mean 0.02, SD 0.04). Most uncertainty-related posts were categorized as having below-median popularity and engagement. The uncertainty-related post with the highest engagement was a question about kidney issues and telomere length posted on Facebook community group by a parent of a child with TBDs, which generated 12 comments from 6 unique users, including a self-identified medical expert. The nonnormal distribution combined with low (<20) frequency in cross-tabulation groups made it ineffective to analyze the relationships between the presence of social support and popularity or engagement (Multimedia Appendix 5).

In the Facebook community group, posts were created by 67 unique individuals, representing 35.8% (183/511) of all group members. Frequency per user was positively skewed toward lower numbers (range 1-94 posts and median 3 posts), and the majority of post creators (343/511, 67.1%) generated ≤5 posts. Although Team Telomere rarely posted directly on the Facebook community group (8/511, 1.6% posts), the top 2 post creators (156/511, 30.5% posts) were identified as White, female, parents of children affected by DC who were also group moderators for Team Telomere. After removing the moderators, the remaining median post frequency was 3 posts per user, with 22.3% (114/511) of the users creating only a single post.

### Sentiment

The majority of posts (2208/2760, 80%) on all social media types were categorized as positive sentiment. Negative sentiment was rarely expressed and was more likely to be expressed on Facebook compared with Twitter (χ^2^_1_=45.4; *P*<.001). Uncertainty-related posts demonstrated a similarly high frequency of positive sentiment across all social media types (Facebook community group: 433/511, 84.7%; Facebook main page: 1495/1815, 82.37%; and Twitter: 328/434, 75.6%; Multimedia Appendix 6).

## Discussion

### Principal Findings

In this study, we explored the use of TBD social media to express health-related uncertainty. We found that uncertainty was a frequent focus of TBD social media across platforms but was primarily limited to scientific issues, requests for informational support, and offers of emotional support, with most posts generated by White, female, English-speaking parents of children with TBDs. These findings are in keeping with other research on rare disease internet-based communities, which found that post content focused on biomedical questions and emotional support provision [63] and was frequently created by White, female users [40,63-65].

The high frequency of uncertainty-related posts on TBD social media created by female caregivers suggests a potentially higher burden of uncertainty management among mothers, which is in agreement with the extensive literature documenting the psychosocial burden of childhood illness on female caregivers [66-68]. However, the observed demographics of TBD social media users may also be an artifact of greater social media engagement among this group, as previous research suggests that female users frequently rely on internet-based communities for navigating uncertainty related to motherhood and other sex-specific health topics [69,70]. Additional research is needed to investigate the relative burden of medical uncertainty among female care providers and to understand the potential barriers to internet-based community formation for users outside this identity group.

Despite the multiplicity of identified uncertainty sources, issues, management, and social support strategies, we found that scientific uncertainty, informational support, and emotional support were the predominant features of uncertainty-related posts on TBD social media. The high frequency of scientific uncertainty issues across platforms suggests that limited scientific and medical knowledge is a salient concern for the TBD community. Gaps in scientific knowledge likely contribute to the focus on probability as a source of uncertainty in TBD social media posts, especially concerning matters such as prognosis, diagnosis, and symptom experiences. Informational support was the most common form of social support in uncertainty-related posts overall, which is in line with other studies showing information seeking as the principal motivator for participation in disease-specific social media [24,26,71-73]. The high frequency of emotional support suggests the potential for TBD social media to enable uncertainty management through person-focused strategies, such as community building, networking, and relationship formation, as seen in other rare disease contexts [24,72]. In addition, evidence of positive asynchronous internet-based communication as a form of “cybertherapy” [32,44] suggests that the emotionally supportive culture of TBD social media may provide psychological benefits for peers, even without explicit conversations about the personal burden of uncertainty. In addition, items coded as emotional support (eg, emoji hearts) that appeared in response to a variety of uncertainty-related content may have communicated multiple forms of support (eg, care, approval, agreement, or affinity) and may be a common reaction to intractable sources of uncertainty, such as probabilistic and scientific unknowns surrounding TBDs. Further exploration of the complex, dynamic, and potentially interactive relationships between social support and uncertainty on social media may be a fruitful area of investigation for future studies.

Given the evidence of the high psychosocial burden of personal uncertainty in similar rare disease contexts [18,36,74,75], it is surprising that the mental and emotional impacts of uncertainty appeared infrequently in TBD social media discussions. When these topics did arise, they were more likely to appear on Twitter content generated by Team Telomere, as opposed to within the conversations of individual users. In the Facebook community group, the impact of uncertainty on personal life was commonly presented in terms of practical issues and focused on ordering uncertainty, such as providing lists of symptoms, organizing information and screening schedules, and triaging problems. This suggests that despite the frequent focus on personal uncertainty issues by Team Telomere, most individual users engaged with TBD social media to troubleshoot and strategize practical issues, rather than to discuss the impact of uncertainty on areas of psychosocial well-being, such as personal identity, goals, or values. This is also reflected in the positive sentiment valence and rare expression of negative emotion on TBD social media, which suggest that social media may not be perceived as a “safe space” for exploring personal topics beyond surface-level stressors [23]. Future research is needed to investigate the shortcomings of social media for expressing personal uncertainty and painful emotions and may highlight a need for psychosocial support to fill this gap in TBD community resources.

Our finding that uncertainty-related support varied by platform could be explained by differences in the structure and expectations of engagement inherent to Twitter compared with the Facebook community group. The predominance of emotional support and greater overall user engagement in the Facebook community group suggests that internet-based platforms structured for mutual conversational exchange may have the most utility for psychosocial support delivery. In addition, the Facebook community group may have encouraged more community participation owing to user familiarity with the platform and its explicit creation for supportive internet-based connection in the context of COVID-19 isolation. Similarly, the nature of the Twitter platform, which is limited to one-way communication streams, suggests that uncertainty management and social support on Twitter would be limited to information provision. However, recent research indicates that Twitter retweets and endorsements may be effective methods for receiving and providing emotional support [76]. The formation of the Facebook community group and the use of Twitter to encourage community activities (eg, webinars and internet-based meetups) underscores the potential of these platforms in person-focused uncertainty management, but additional research is required to evaluate the capacity of TBD social media to build health-promoting personal relationships.

Although we found substantial potential for social media to deliver support for uncertainty management, analysis of engagement rates demonstrated that the primary function of TBD social media was a “drop-in” source of information. Although the Facebook community group included some multilevel, ongoing conversations, an analysis of posts within this group revealed that most user engagement was limited to single posts, suggesting quick check-ins or requests for answers to targeted questions, not ongoing social connection. Although low engagement may suggest limited supportive utility of TBD social media, findings from previous research with young adults with cancer showed that support delivered via social media benefited a variety of users, including those actively seeking deep connections, those seeking information only, and those who do not actively participate but frequently observe the conversation of others (eg, “lurkers”) [77]. As suggested by other research, any benefit from engagement with social media likely varies over time and may be most pronounced during experiences of novelty or discrepancy in diagnosis, treatment, or prognosis [28,48,63]. The uncertainty-related post that generated the highest engagement involved the participation of a medical expert, suggesting a desire among TBD social media users to engage with clinicians on internet-based platforms that facilitate reciprocal information exchange, including both synchronous (eg, internet-based group meetings) and asynchronous (eg, post exchanges) formats. Further research is needed to understand the motivations, perceived benefits, and perceived barriers to participation in TBD internet-based support platforms, including the perspectives of patients, caregivers, and medical providers.

### Limitations

The limitations of our study include the use of social media data, which biases our sample toward active social media users who may have higher levels of distress [64], greater disenchantment with medical care [78], or lower perceived social support [79] compared with patients with TBDs and their families who do not actively use social media. Demographic analysis revealed that our sample of posts was generated primarily by White females, parents of patients with TBDs, or representatives of Team Telomere. This limited the generalizability of our findings. In addition, our use of social media posts, rather than content creators, as the unit of analysis precludes the observation of the longitudinal impacts of social media participation on uncertainty management. Furthermore, our findings allow us to infer the presence of uncertainty management strategies on social media but not the motivations for or effects of these activities.

In addition, our data were limited to social media that was actively moderated by Team Telomere. This moderation activity, which included removing posts that were inappropriate or scientifically inaccurate, likely decreased the presence of medical misinformation compared with unmoderated social media content. The moderation of posts by Team Telomere could also have impacted the range and authenticity of social and emotional expression owing to social desirability bias. This is in keeping with recent research challenging the assumption that the privacy and anonymity of internet-based environments decreases the likelihood of social desirability compared with in-person interactions [80,81]. In addition, we did not access the private Facebook community group maintained by Team Telomere described as “where we share detailed and private medical information” [57], which may contain additional uncertainty-related posts and a wider range of social and emotional expression. Limiting ourselves to social media owned and maintained by Team Telomere also prevented us from discerning the perspectives of individuals affected by TBD who lacked knowledge of or who chose not to engage with Team Telomere.

Finally, our study was limited by the occurrence of the COVID-19 pandemic, first mentioned in Team Telomere social media on February 28, 2020, which may have changed the nature of uncertainty-related conversations or social support in that portion of our data timeline (June 6, 2019, to December 7, 2021). To test the impact of this, we included available posts (Twitter and Facebook main page) from 1 year before the pandemic and tested the difference. Greater frequencies of uncertainty-related posts after COVID-19 suggest that the pandemic may have increased the expression of uncertainty on TBD-related social media, thus limiting the applicability of our findings to other time points (Multimedia Appendix 7).

### Conclusions

This study found the frequent use of disease-specific social media for the discussion and management of uncertainty in TBDs. Uncertainty-related posts appeared across all TBD social media platforms and communicated a burden of multiple, often interacting sources and issues of uncertainty, particularly focused on scientific knowledge gaps and the desire to predict health outcomes. Posts also indicated multiple uncertainty management attributes, with a focus on information-seeking and community-building approaches. Uncertainty-related posts frequently co-occurred with social support, primarily emotional and informational. Female parents were most often the creators of uncertainty-related posts on TBD social media, suggesting a potentially higher burden of uncertainty management in this population. Overall, social media provided access to a positive emotional environment and frequent information exchange but was limited in the type and depth of uncertainty-related discussions. Despite these limitations, our findings suggest that social media is a useful lens for researching and understanding the experience of uncertainty in TBDs and holds potential as a tool for uncertainty management. Future research is needed to further explore the experience of medical uncertainty in TBDs and to determine the usefulness of TBD-related social media as a tool for improving mental health and quality of life outcomes in this context.

## Appendix

Multimedia Appendix 1 Criteria for identification of posts for inclusion in qualitative uncertainty analysis.

Multimedia Appendix 2 Social media study codebook.

Multimedia Appendix 3 Emoji dictionary.

Multimedia Appendix 4 Frequency of social support by support type and direction.

Multimedia Appendix 5 Engagement and popularity by platform.

Multimedia Appendix 6 Sentiment by post type.

Multimedia Appendix 7 COVID-19 impact summary.

## Abbreviations

DC: dyskeratosis congenita
TBD: telomere biology disorder

## Data Availability

In compliance with the National Institutes of Health data management and sharing policy, data, analysis code, and research materials are available upon reasonable request from the corresponding author.
