# Supplementary material for: The Use of Social Media to Express and Manage Medical Uncertainty in Dyskeratosis Congenita: Content Analysis
Source: JMIR Infodemiology. 2024 Jan 15;4:e46693. doi: 10.2196/46693 (PMC10825764; doi:10.2196/46693)
Supplement: Multimedia Appendix 1 [file infodemiology_v4i1e46693_app1.docx]

**Multimedia Appendix 1**

**Criteria for Identification of Posts for Inclusion in Qualitative Uncertainty Analysis**

1. An expression of ignorance or an attempt to engage in uncertainty management through a request for information or feedback (e.g., asking a question or soliciting confirmation of the “normality” of an experience, patient-as-scientist)

**Examples**

Why do you have to do bone marrow testing every year for DC?

Who is taking danazole? Pros/cons, what were your counts before and after starting?

2. Acknowledgment of ignorance due to challenges to one’s prior expectations of a) risk (e.g., previously low risk activities becoming higher risk activities; concerns about injury, illness, or mortality arising from previously low-risk behavior) or b) likelihood of achievements (e.g., celebration of continued survival, relief, and life event milestones; we weren’t sure we would make it this far)

Examples

*It was fun, but I came home from this trip with pneumonia.*

*When you have an itch on your nose and you forget your platelets are 24. . .*

She just has a wee cold but has been off all week.

*I can't believe how much they weigh(ed as premature births) and made it!*

*on my way to the clinic…day 26+*

*Happy 8th bone marrow transplant anniversary!*

3. Uncertainty management strategies offered by Team Telomere (e.g., Family Chats, TT Tuesday check ins, yoga, “celebrating rare”/community-building, sharing research results or news articles)

Examples

*Similar pages have been created by other rare disease groups during this time of increased isolation.*

*A place of no judgement, come as you are and begin from where you are.*

*We are constantly trying to find new ways to improve and give you the most support possible, here are a few resources*

4. Uncertainty management strategy (other than direct questioning/information seeking captured in #1) being enacted by a community member (e.g., reminiscence, expressing shared identity, patient-as-expert/sharing “lessons-learned”, survivor stories, and parables)

Examples

*Came across this photo and had to share. It simultaneously brought me a smile and a tear.*

It's NIH document day! / My favorite! 😀. I have some test kits on my dining room table.

fitting for us all: it wasn't the trauma that made you strong, kinder, and more compassionate. It's how you handled it. That credit is yours
