# Supplementary material for: The Use of Social Media to Express and Manage Medical Uncertainty in Dyskeratosis Congenita: Content Analysis
Source: JMIR Infodemiology. 2024 Jan 15;4:e46693. doi: 10.2196/46693 (PMC10825764; doi:10.2196/46693)
Supplement: Multimedia Appendix 2 [file infodemiology_v4i1e46693_app2.docx]

**Multimedia Appendix 2**

**Uncertainty in DC Social Media Codebook**

| **#** | **Code** |  | **Definition** |
| --- | --- | --- | --- |
| 1 | Social Support | 1.1 Emotional | Evidence of giving or receiving positive emotional support |
|  |  | 1.2 Instrumental | Evidence of giving or receiving tangible aid or services |
|  |  | 1.3 Appraisal | Evidence of giving or receiving information or advice useful for self-evaluation |
|  |  | 1.4 Informational | Evidence of giving or receiving information, advice, or education useful for problem-solving, learning skills, or gaining knowledge |
|  |  | 1.5 Unsupportive reaction | If request for support is made, the response received is not intended to be supportive |
| 2 | Source of Uncertainty | 2.1 Ambiguity | Evidence of difficulty in interpreting or understanding due to ambiguous information features. |
|  |  | 2.2 Complexity | Evidence of difficulty in interpreting or understanding due to complex information features. |
|  |  | 2.3 Probability | Evidence of difficulty in interpreting or understanding due to predictive information features. |
| 3 | Issues of Uncertainty | 3.1 Personal | Expression of uncertainty about aspects of personal life |
|  |  | 3.2 Practical | Expression of uncertainty about logistical issues related to healthcare |
|  |  | 3.3 Scientific | Expression of uncertainty about disease processes, management |
| 4 | Uncertainty Management Attributes | 4.1 Ignorance-Focused | Evidence of actions that could reduce or eliminate ignorance |
|  |  | 4.2 Uncertainty-Focused | Evidence of actions that could increase or decrease consciousness of ignorance |
|  |  | 4.3 Response-Focused | Evidence of actions that could mitigate or palliate psychological response to ignorance |
|  |  | 4.4 Person-Focused | Evidence of actions that could target the intra- and inter-personal impact of uncertainty on relationships |
| 5 | Locus of Uncertainty | 5.1 Own Mind | The uncertainty is situated in the mind of the person generating the post; the person generating the post is responding to their own uncertainty. |
|  |  | 5.2 Another’s Mind | The uncertainty is situated in the mind of another person; the person generating the post is responding to another’s uncertainty. |
| 6 | Uncertainty Quotient | 6.1 Increasing | Attributes of an uncertainty-related post or conversation (series of posts, comments, and responses) that could result in increasing uncertainty. |
|  |  | 6.2 Decreasing | Attributes of an uncertainty-related post or conversation (series of posts, comments, and responses) that could result in decreasing uncertainty. |
| 7 | Expression of Uncertainty | 7.1 Direct | Post in which expression of uncertainty is clearly articulated as a question or comment. |
|  |  | 7.2 Indirect | Post in which expression of uncertainty may be implicit, but is not clearly articulated |
| 8 | Uncertainty Tolerance Capacities | 8.1 Uncertainty Tolerance Capacity | Evidence of attributes that could facilitate adaptation into a state of balance from disequilibrium (which ultimately can allow individual to overcome the overwhelm of uncertainty and move forward) |
| 9 | Sentiment | 9.1 Positive | Expression of thoughts, feelings, or emotions that may could indicate experience of positive emotion, resilience, flourishing, or thriving |
|  |  | 9.2 Negative | Expression of thoughts, feelings or symptoms that could indicate experience of negative emotion or mental health challenges such as anxiety or depression |
