# Supplementary material for: The Use of Social Media to Express and Manage Medical Uncertainty in Dyskeratosis Congenita: Content Analysis
Source: JMIR Infodemiology. 2024 Jan 15;4:e46693. doi: 10.2196/46693 (PMC10825764; doi:10.2196/46693)
Supplement: Multimedia Appendix 3 [file infodemiology_v4i1e46693_app3.docx]

**Multimedia Appendix 3**

**Emoji Dictionary**

| **Image** | **Description** | **Category** | **Sentiment** |
| --- | --- | --- | --- |
| 😒 | Unamused face | EmoAngry | Negative |
| 😬 | Grimacing face | EmoAngry | Negative |
| 😤 | Face with steam | EmoAngry | Negative |
| 😡 | Pouting face | EmoAngry | Negative |
| 😠 | Angry face | EmoAngry | Negative |
| 💢 | Anger | EmoAngry | Negative |
| 🤗 | Hugs | EmoCare | Positive |
| 😅 | Smiling face with tears | EmoHaha | Positive |
| 😆 | Grinning face | EmoHaha | Positive |
| 🤣 | Rolling on the floor laughing | EmoHaha | Positive |
| 😂 | Lauging with tears | EmoHaha | Positive |
| 🙃 | Upside down face | EmoHaha | Positive |
| 😉 | Winking face | EmoHaha | Positive |
| 😊 | Smiling face with smiling eyes | EmoHaha | Positive |
| 😅 | Smiling face with tears | EmoHaha | Positive |
| 😋 | Yummy face | EmoHaha | Positive |
| 😛 | Face with tongue | EmoHaha | Positive |
| 😜 | WInking face with tongue | EmoHaha | Positive |
| 🤪 | Zanny face | EmoHaha | Positive |
| 😝 | Squinting face with tongue | EmoHaha | Positive |
| 🤭 | Face with hand in mouth | EmoHaha | Positive |
| 😏 | Smirking face | EmoHaha | Positive |
| 🤞 | Crossed fingers | EmoHope | Positive |
| 🙏 | Praying hands | EmoHope | Positive |
| 🤔 | Thinking face | EmoHuh | Neutral |
| 🤨 | Face with raised eyebrow | EmoHuh | Neutral |
| 😔 | Pensive face | EmoHuh | Neutral |
| 😕 | Confused face | EmoHuh | Neutral |
| 🤷‍♀️ | Shrugging shoulders | EmoHuh | Neutral |
| 🙂 | Slightly smiling face | EmoLike | Positive |
| 😀 | Smiling face | EmoLike | Positive |
| 😃 | Smiling face with big eyes | EmoLike | Positive |
| 😄 | Smiling face with smiling eyes | EmoLike | Positive |
| 😁 | Beaming face with smiling eyes | EmoLike | Positive |
| 😇 | Smiling face with halo | EmoLike | Positive |
| 😎 | Smiling face with sunglasses | EmoLike | Positive |
| 🧐 | Face with monocle | EmoLike | Positive |
| ☺ | Smiling face | EmoLike | Positive |
| 👍🏼 | Thumbs Up | EmoLike | Positive |
| 🥰 | Smiling face with hearts | EmoLove | Positive |
| 😍 | Smiling face with heart eyes | EmoLove | Positive |
| 🤩 | Star-struck | EmoLove | Positive |
| 😘 | Face blowing kiss | EmoLove | Positive |
| 😗 | Kissing face | EmoLove | Positive |
| 😚 | Kissing face with closed eyes | EmoLove | Positive |
| 😙 | Kissng face with smiling eyes | EmoLove | Positive |
| 💋 | Kiss | EmoLove | Positive |
| 💌 | Love letter | EmoLove | Positive |
| 💘 | Heart with arrow | EmoLove | Positive |
| 💝 | HEart with ribbon | EmoLove | Positive |
| 💖 | Sparking heart | EmoLove | Positive |
| 💗 | Growing heart | EmoLove | Positive |
| 💓 | Beating heart | EmoLove | Positive |
| 💞 | Revolving heart | EmoLove | Positive |
| 💕 | Two hearts | EmoLove | Positive |
| 💟 | Heart decoration | EmoLove | Positive |
| ❣ | Heart exclamation | EmoLove | Positive |
| ❤️‍🔥 | Heart on fire | EmoLove | Positive |
| ❤️‍🩹 | Mending heart | EmoLove | Positive |
| ❤ | Red heart | EmoLove | Positive |
| 🧡 | Orange heart | EmoLove | Positive |
| 💛 | Yellow heart | EmoLove | Positive |
| 💚 | Green heart | EmoLove | Positive |
| 💙 | Blue heart | EmoLove | Positive |
| 💜 | Purple heart | EmoLove | Positive |
| 🤎 | Brown heart | EmoLove | Positive |
| 🖤 | Black heart | EmoLove | Positive |
| 🤍 | White heart | EmoLove | Positive |
| 🤟 | Love you | EmoLove | Positive |
| 😷 | Face with mask | EmoSad | Negative |
| 🤒 | Face with thermometer | EmoSad | Negative |
| 🤕 | Face with bandage | EmoSad | Negative |
| 😟 | Worried face | EmoSad | Negative |
| 🙁 | Slightly frowning face | EmoSad | Negative |
| ☹ | Frowning face | EmoSad | Negative |
| 🥺 | Begging face | EmoSad | Negative |
| 😦 | Frowning face with open mouth | EmoSad | Negative |
| 😧 | Anguished face | EmoSad | Negative |
| 😨 | Fearful face | EmoSad | Negative |
| 😰 | Anxious face with sweat | EmoSad | Negative |
| 😥 | Sad but relieved face | EmoSad | Negative |
| 😢 | Crying face | EmoSad | Negative |
| 😱 | Screaming face | EmoSad | Negative |
| 😞 | Disappointed face | EmoSad | Negative |
| 💔 | Broken heart | Emo Sad | Negative |
| 🧬 | DNA | EmoSci | Positive |
| 🔬 | Microscope | EmoSci | Positive |
| 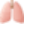   \| 🫁 \| \| --- \| | Lung | EmoSci | Positive |
| 🦠 | amoeba? | EmoSci | Positive |
| 🧫 | Petri dish | EmoSci | Positive |
| 😪 | Sleepy face | EmoTired | Neutral |
| 😴 | Sleeping face | EmoTired | Neutral |
| 😩 | Weary face | EmoTired | Neutral |
| 😫 | Tired face | EmoTired | Neutral |
| 🥱 | Yawning face | EmoTired | Neutral |
| 💤 | Zzz | EmoTired | Neutral |
| 😮 | Face with open mouth | EmoWow | Positive |
| 😯 | Hushed face | EmoWow | Positive |
| 😲 | Astonished face | EmoWow | Positive |
| 😳 | Flushed face | EmoWow | Positive |
| 🥳 | Partying face | EmoYay | Positive |
| 👍 | Thumbs up | EmoYay | Positive |
| 👏 | Clapping hands | EmoYay | Positive |
| 🎆 | Fireworks | EmoYay | Positive |
| 🎇 | Sparkler | EmoYay | Positive |
| 🧨 | Firecracker | EmoYay | Positive |
| ✨ | Sparkles | EmoYay | Positive |
| 🎈 | Balloon | EmoYay | Positive |
| 🎉 | Party popper | EmoYay | Positive |
| 🎊 | Confetti ball | EmoYay | Positive |
| 🎁 | Wrapped gift | EmoYay | Positive |
| 🎖 | Military medal | EmoYay | Positive |
| 🏆 | Trophy | EmoYay | Positive |
| 🏅 | Sports medal | EmoYay | Positive |
| 🥇 | Gold medal - first position | EmoYay | Positive |
| 🥈 | Silver medal - second position | EmoYay | Positive |
| 🥉 | Bronze medal - third position | EmoYay | Positive |
| 🎂 | Birthday cake | EmoYay | Positive |
| !! | Exclamation marks | EmoYay | Positive |
| 🙌 | High Five | EmoYay | Positive |
| 👊 | Fist Bump | EmoYay | Positive |
| 🥁 | Drum Roll | EmoYay | Positive |
