# Supplementary material for: The Use of Social Media to Express and Manage Medical Uncertainty in Dyskeratosis Congenita: Content Analysis
Source: JMIR Infodemiology. 2024 Jan 15;4:e46693. doi: 10.2196/46693 (PMC10825764; doi:10.2196/46693)
Supplement: Multimedia Appendix 4 [file infodemiology_v4i1e46693_app4.docx]

Multimedia Appendix 4

Figure 1. Frequency of social support by support type (appraisal support values were omitted because of low frequencies; range 0%-0.6%) and direction for (A) uncertainty-related posts by platform (n=1489), (B) Facebook community group uncertainty-related post subtype (uncertainty post subtypes included posts coded as uncertainty-related primary posts [n=42], uncertainty-related comments [n=114], and posts that were not uncertainty related but were contained within a thread where ≥1 other posts were uncertainty related [n=127]; n=283), (C) Facebook community group uncertainty issue subtype (uncertainty issue and source subtypes were not mutually exclusive, were coded only for uncertainty-related posts and comments [n=156], and did not include non–uncertainty-related posts contained within uncertainty-related threads [n=127]; n=156), and (D) Facebook community group uncertainty source subtype (uncertainty issue and source subtypes were not mutually exclusive, were coded only for uncertainty-related posts and comments [n=156], and did not include non–uncertainty-related posts contained within uncertainty-related threads [n=127]; n=156).


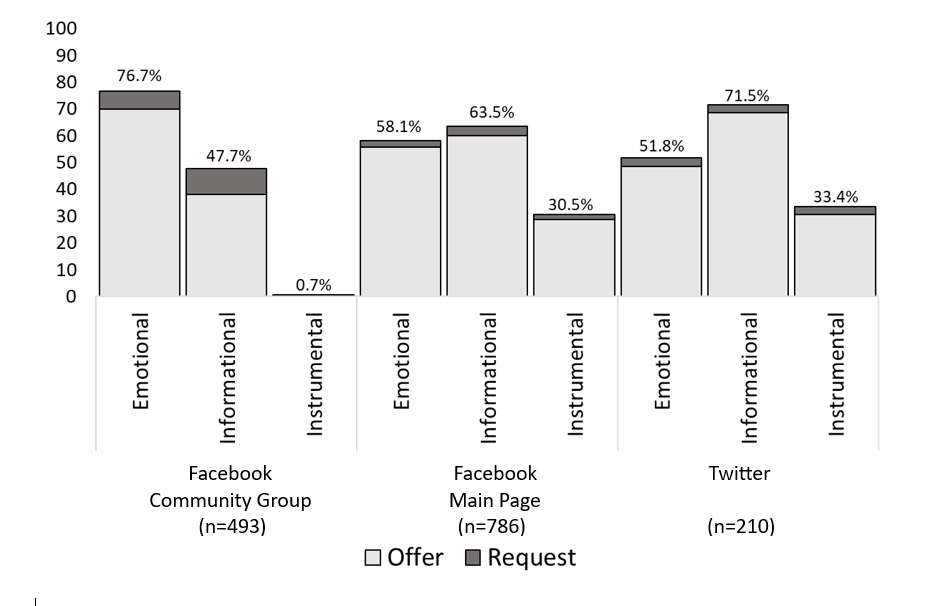
**A.**

Facebook Facebook Twitter

Community Group Main Page

(n=493) (n=786) (n=210)

**B.**


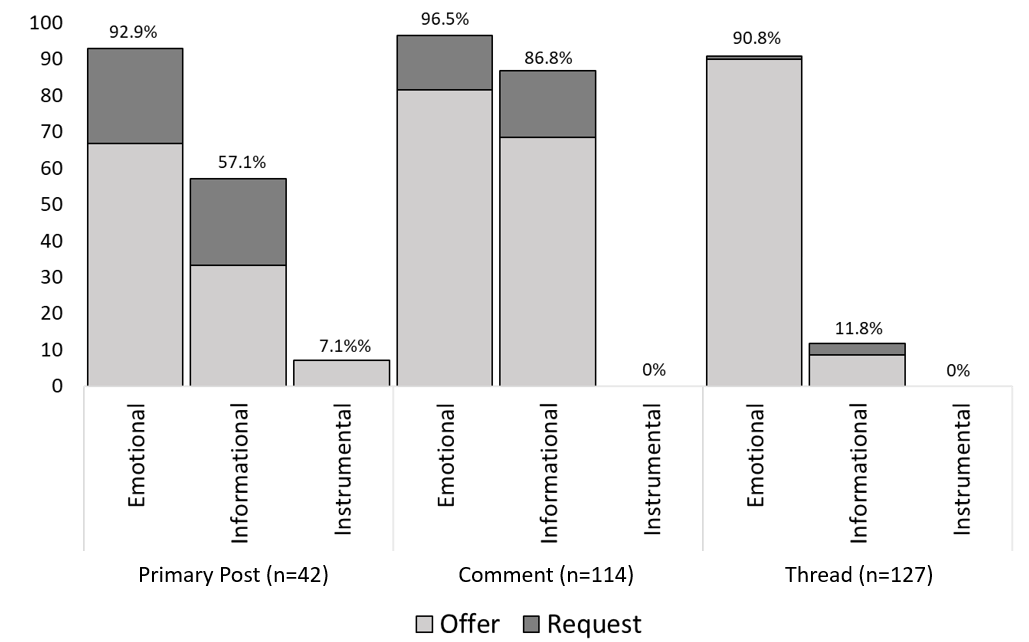


**C.**


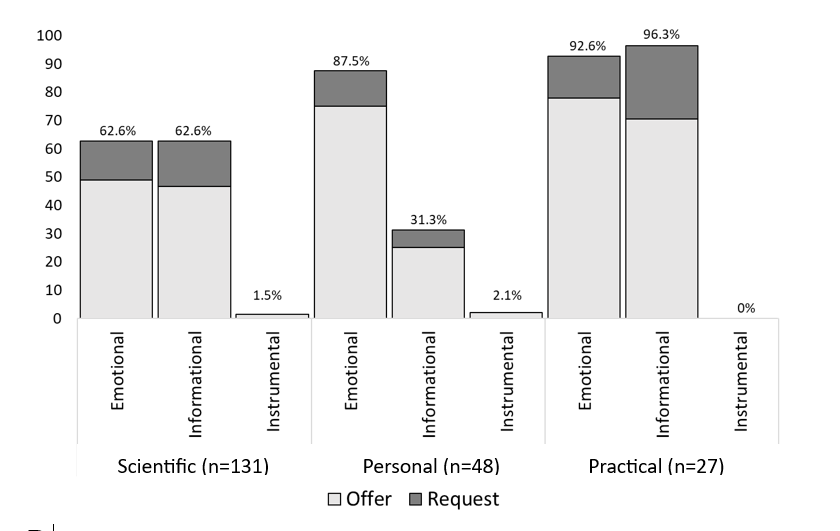


D.


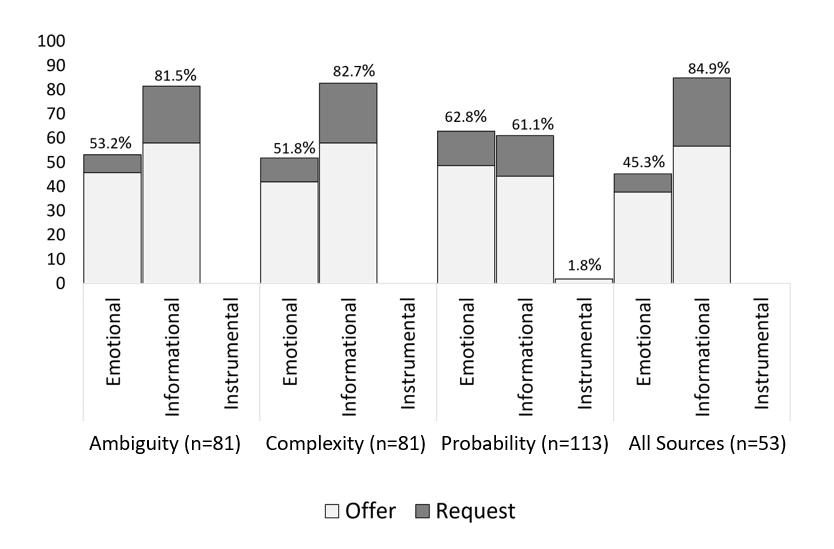


n

¹Appraisal support values are omitted due to low frequencies (range 0 – 0.6%).

²Uncertainty post subtypes included posts coded as uncertainty-related primary posts (n=42), uncertainty-related comments (n=114), and posts that were not uncertainty-related, but were contained within a thread where one or more other posts were uncertainty-related (n=127).

³Uncertainty issue and source subtypes were not mutually exclusive, were coded only for uncertainty-related posts and comments (n=156), and do not include non-uncertainty-related posts contained within uncertainty-related threads (n=127).
