# Supplementary material for: The Use of Social Media to Express and Manage Medical Uncertainty in Dyskeratosis Congenita: Content Analysis
Source: JMIR Infodemiology. 2024 Jan 15;4:e46693. doi: 10.2196/46693 (PMC10825764; doi:10.2196/46693)
Supplement: Multimedia Appendix 5 [file infodemiology_v4i1e46693_app5.docx]

Multimedia Appendix 5

Table 3S. Popularity and Engagement by social media type.

|  | **FB COMMUNITY GROUP** **(N=511)** | **FB MAIN PAGE(N=1815)** | **Twitter** **(N=434)** |
| --- | --- | --- | --- |
|  | *Mean (SD), Median (min-max)* | *Mean (SD), Median (min-max)* | *Mean (SD), Median (min-max)* |
| Popularity¹ | 4.0 (7.47), 1.0 (0-55) | 5.8 (11.26), 1.0 (0-151) | 28.79 (77.21), 13.0 (0-1147) |
| Engagement² | 4.29 (2.98), 3.0 (2-29) | 4.17 (5.08), 3.0 (2-110) | 24.97 (73.95), 11.0 (0-1138) |
|  | *N posts (%)* | *N posts (%)* | *N posts (%)* |
| Popularity > Median | 410 (80.2%) | 1329 (73.2%) | 225 (51.8%) |
| Engagement > Median | 475 (92.9%) | 1262 (69.5%) | 351 (80.9%) |
| ¹Popularity = Sum(Comments, Likes, Shares)  ²Engagement (Twitter) = Sum(Detail Expands, Profile Visits, Link Clicks, Video Views); Engagement (Facebook) = Sum(Conversations, Voices, Depth) | | | |
