# Supplementary material for: The Use of Social Media to Express and Manage Medical Uncertainty in Dyskeratosis Congenita: Content Analysis
Source: JMIR Infodemiology. 2024 Jan 15;4:e46693. doi: 10.2196/46693 (PMC10825764; doi:10.2196/46693)
Supplement: Multimedia Appendix 6 [file infodemiology_v4i1e46693_app6.docx]

Multimedia Appendix 6

Emotional sentiment of all posts (N=2760) by social media type.

|  | **FB COMMUNITY GROUP** **(N=511)** | **FB MAIN PAGE(N=1815)** | **TWITTER** **(N=434)** |  |
| --- | --- | --- | --- | --- |
|  | *N posts (%)* | *N posts (%)* | *N posts (%)* |  |
| **Sentiment (Emoji Only)** |  |  |  |  |
| Negative | 11 (2%) | 30 (2%) | 0 (0%) |  |
| Positive | 396 (78%) | 1369 (75%) | 314 (72%) |  |
| Neutral | 32 (6%) | 107 (6%) | 40 (9%) |  |
| Uncategorized | 72 (14%) | 309 (17%) | 80 (19%) |  |
| **Sentiment (Emoji + Keyword)** | | | | |
| Negative | 42 (8.2%) | 101 (5.9%) | 7 (1.6%) |  |
| Positive | 427 (83.6%) | 1488 (82.7%) | 329 (75.8%) |  |
| Uncategorized | 42 (8.2%) | 226 (12.4%) | 98 (22.6%) |  |

Table 5S. Emoji frequency of all posts (N=2760) by social media type.

|  | **FB COMMUNITY GROUP** **(N=511)** | **FB MAIN PAGE(N=1814)** | **Twitter** **(N=434)** |
| --- | --- | --- | --- |
|  | *N posts (%)* | *N posts (%)* | *N posts (%)* |
| **Emoji Type** |  |  |  |
| Love | 241 (47.2%) | 1014 (55.9%) | 11 (2.5%) |
| Like | 213 (41.7%) | 864 (47.6%) | 311 (72.7%) |
| Care | 53 (10.4%) | 86 (4.7%) | 0 (0%) |
| Haha | 22 (4.3%) | 52 (2.9%) | 2 (0.5%) |
| Yay | 14 (2.7%) | 47 (2.6%) | 6 (1.4%) |
| Science | 1 (0.2%) | 23 (1.3%) | 10 (2.3%) |
| Sad | 11 (2.2%) | 29 (1.6%) | 0 (0%) |
| Wow | 7 (1.4%) | 19 (1.1%) | 0 (0%) |
| Hope | 3 (0.6%) | 6 (0.3%) | 1 (0.2%) |
| Huh | 4 (0.8%) | 2 (0.1%) | 0 (0%) |
| Tired | 0 (0%) | 1 (0.1%) | 0 (0%) |
| Angry | 0 (0%) | 0 (0%) | 0 (0%) |
| *Uncategorized* | *27 (5.3%)* | *105 (5.8%)* | *38 (8.8%)* |
