# Supplementary material for: The Use of Social Media to Express and Manage Medical Uncertainty in Dyskeratosis Congenita: Content Analysis
Source: JMIR Infodemiology. 2024 Jan 15;4:e46693. doi: 10.2196/46693 (PMC10825764; doi:10.2196/46693)
Supplement: Multimedia Appendix 7 [file infodemiology_v4i1e46693_app7.docx]

**Multimedia Appendix 7**

**COVID-19 Impact Summary**

Frequency results did not vary by timing pre- or post-COVID for social support (Χ²=0.31, DF = 1, p=0.58), but did vary for uncertainty-related posts, with more uncertainty-related posts appearing after COVID (Χ²=65.2, DF = 1, p<0.0001). Some variation was observed by social support subtype, with information and emotional support frequency slightly higher post-COVID compared to pre-COVID (Χ²=4.5, DF = 1, p=0.03 and Χ²=7.7, DF = 1, p=0.005, respectively). These analyses were repeated removing the FB COMMUNITY GROUP data, as FB COMMUNITY GROUP was formed in response to COVID. The pre- and post-COVID differences remained, with no difference for social support (Χ²=0.03, DF = 1, p=0.87), and significant difference for uncertainty-related posts, with more appearing after COVID (Χ²=48.7, DF = 1, p<0.0001). In social support subtypes, informational support frequency was higher and emotional support was lower post-COVID (Χ²=48.7, DF = 1, p<0.0001 and Χ²=26.6, DF = 1, p<0.0001, respectively).

These results were further explored by removing COVID-related posts (N=432) and re-running the analysis. After removing COVID-related posts there was no difference in social support frequency pre- and post-COVID, but differences in uncertainty-related posts remained, with uncertainty related posts more frequent post-COVID (72%) than pre-COVID (28%) (X²=42.8, DF=1, p<0.0001).
